# Watch and Wait Management of Inactive Cystic Echinococcosis – Does the Path to Inactivity Matter – Analysis of a Prospective Patient Cohort

**DOI:** 10.1371/journal.pntd.0005243

**Published:** 2016-12-19

**Authors:** Marija Stojkovic, Kerstin Daniela Rosenberger, Franziska Steudle, Thomas Junghanss

**Affiliations:** Heidelberg University Hospital, Section Clinical Tropical Medicine, Heidelberg, Germany; Istituto Superiore di Sanità, ITALY

## Abstract

**Background:**

Overdiagnosis and overtreatment are rarely discussed in the context of NTDs despite their relevance for patients under the care of health services with limited resources where the risks of therapy induced complications are often disproportionate to the benefit. The advantages of cyst staging-based management of patients with cystic echinococcosis (CE) are not yet fully explored. Questions are: Do inactive cysts (CE 4 and CE 5) need treatment and is there a difference between cysts which reach CE4 and CE5 naturally or by benzimidazole therapy?

**Methodology/Principal findings:**

Analysis of long-term follow-up data from a prospective CE patient cohort of 223 patients of a national clinical center for echinococcosis. The event of interest “relapse” was defined as the reversal of a cyst from an inactive stage (CE4, CE5) back to an active stage. The watch &wait (ww) group included 30 patients with 46 inactive cysts who never received medical treatment. The benzimidazole-treated (med) group included 15 patients with 17 cysts. There was no relapse in the ww-group whereas 8/17 cysts showed relapse within 18 months after treatment in the med-group. Loss to follow-up was 15.5%.

**Conclusions:**

Data from the watch & wait group impressively show how stable naturally inactivated cysts are in contrast to cysts which reach inactivity through treatment with benzimidazoles. A substantial proportion of patients can be spared from treatment through cyst staging. Cysts which inactivated through a natural course do not relapse with very high likelihood. We recommend follow up of 5 years to confirm the stability of the inactive stage. Cysts driven into inactivity through benzimidazole therapy instead need careful monitoring to identify those which reactivate (around 50% within 18 months). 5 years follow-up appears safe to make a final decision on the need for further monitoring.

## Author Summary

Cystic echinococcosis (CE) is a zoonosis of worldwide distribution causing cystic lesions in the liver and lung. The prevalence of disease is highest in countries with limited resources. Overdiagnosis and overtreatment are increasingly discussed in medical practice not surprisingly also for patients with cystic echinococcosis. In neglected tropical diseases (NTD) this is particularly relevant since most patients are under the care of health services with limited resources where the risk of therapy induced complications are often disproportionate to the benefit of interventions. Our study aims at answering the following Questions: Do inactive cysts (CE 4 and CE 5) need treatment and is there a difference between cysts which reach CE4 and CE5 naturally or by benzimidazole therapy? Results of our study impressively show that a substantial proportion of patients can be spared from any treatment through cyst staging. Cysts which inactivated through a natural course of involution do not relapse with very high likelihood. To rule out unlikely events, we recommend follow up of 5 years to confirm the stability of the inactive stage. Cysts driven into inactivity instead need careful monitoring to identify those which reactivate (around 50% within 18 months). 5 years follow-up appears safe to make a final decision.

## Introduction

Overdiagnosis and overtreatment are increasingly discussed in medical practice not surprisingly also for patients with cystic echinococcosis (CE). In neglected tropical diseases (NTD) this is particularly relevant since most patients are under the care of health services with limited resources where the risk of therapy induced complications are often disproportionate to the benefit of interventions. In high-tech environments conditions which would never have surfaced and bothered a person regularly get unintentionally diagnosed and unnecessarily treated [1].

CE cysts are staged on the basis of the WHO-IWGE ultrasound (US)-based cyst classification [2] into “active” (CE1, CE2), “transitional” (CE3a and CE3b) and “inactive” (CE4, CE5). Magnetic resonance imaging (MRI) reproduces the US-defined features, less so computed tomography (CT) [3–5]. Observations in CE reference centers generated significant evidence for two interesting and very relevant hypotheses: (a) uncomplicated active (CE1 and CE2) or transitional (CE3a and CE3b) CE cysts which progressed without treatment to stage CE 4 and CE 5 do not reactivate and can safely be left untreated, whereas (b) if these cyst stages are induced by albendazole (ABZ) treatment relapse is common and patients need to be followed-up long enough to not miss it.

The objective of this study is to test these two hypotheses in a prospectively followed-up well documented clinical cohort.

## Methods

The Section of Clinical Tropical Medicine at Heidelberg University Hospital runs an interdisciplinary clinic for echinococcosis in cooperation with the Department of Diagnostic and Interventional Radiology with weekly radiological conferences, the Department of Surgery, the Department of Thoracic Surgery, the Interdisciplinary Endoscopy Center, and the Department of Parasitology since 1999. Our unit is a national clinical center for echinococcosis. Patient data is collected prospectively, at every visit of CE patients a defined set of cyst and treatment data are entered into the CE database. The CE patient database contained 223 consecutive patients in February 2014. The database was screened for patients with w&w management or medical treatment and all follow-up data of patients eligible for the study were updated in June 2016.

**Fig 1:**
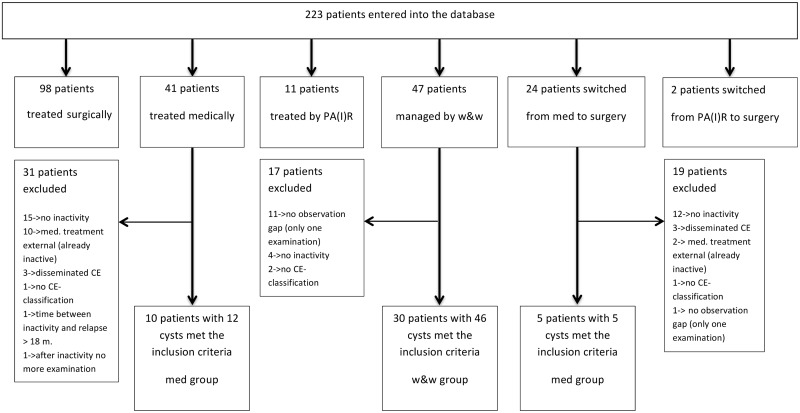
Treatment groups and patient selection of the Heidelberg CE patient cohort.

Of 223 patients entered into the database, 114 patients were male and 109 female, the mean age was 46.14 (2.62–90.79). Further details of the patient cohort are shown in Fig 1.

### Inclusion criteria

#### Watch & wait (ww) group

Patients with single or multiple (maximum 8) inactive CE4 or CE5 cysts who never received medical treatment for CE.

#### Medical treatment (med) group

Patients with single or multiple (maximum 8) active CE cysts which were treated with benzimidazoles for CE and became inactive.

To determine the time to relapse with sufficient accuracy, relapse from CE4 and CE5 to an active cyst stage was only included into the analysis if the time between the visit when the cyst was still inactive and the visit when it relapsed was ≤ 18 months. Patients were followed-up by ultrasound or magnetic resonance imaging. A patient was considered lost to follow-up if he did not complete a minimum follow-up of 5 years or the last follow-up visit was > 24 months ago. Patients with disseminated CE were excluded.

### Ethics statement

The Ethical Board of the University of Heidelberg (S126/2010) approved the analysis of patient data form the CE patient database without additional patient consent. All patient medical data analyzed were anonymized.

### Data analysis

The event of interest “relapse” was defined as the reversal of a cyst from an inactive stage (CE4, CE5) back to an active stage.

Proportion of cyst relapse in the med-group was calculated for each follow-up year since reaching the inactive stage. The numerator consists of the number of cysts with relapse during the respective time window, the denominator is the number of cysts still under observation which are at risk for relapse. A binomial exact 95% confidence interval was calculated for each follow-up year.

In case medical treatment was still on going at the time of the visit, the time at risk started at the end of therapy. If this date did not coincide with a visit to the clinic, the cyst stage from the last visit was considered (last observation carried forward).

In order to account for censoring we performed a time-to-event analysis for the med group. The start of the time at risk for each cyst was defined as the date of the visit when the inactive stage was diagnosed. In case medical treatment was still ongoing at the time of the visit, the time at risk started at the end of therapy. If this date did not coincide with a visit to the clinic, the cyst stage from the last visit was considered (last observation carried forward).

We analyzed time to relapse using the Kaplan-Meier method. Cumulative incidence of relapse was calculated as one minus the Kaplan-Meier estimate.

All analyses were performed using Stata Version 13.1 (StataCorp, College Station, TX) and R software version 3.3.1 [6].

Descriptive results for continuous variables are given as mean (min, max) if not stated otherwise.

## Results

### Baseline data

The selection of study patients of the Heidelberg CE patient cohort is displayed in Fig 1.

Forty-five patients of the Heidelberg CE patient cohort fulfilled the inclusion criteria. Thirty patients with 46 cysts were included in the ww-group, and 15 patients with 17 cysts into the med-group. The mean age of ww-patients was 41.5 (8–69), 18 patients were male, 12 patients female, mean follow-up for ww-patients was 5.4 (0.5–10.9) years. 6 patients were lost to follow-up in the ww-group. The mean age of med-patients was 35.8 (13–65), 4 patients were male, 11 patients female, mean follow-up of med-patients was 7.3 (1.5–11.8) years. One patient was lost to follow-up in the med-group. Patients were from the following countries: Turkey: 16, countries of the former Yugoslavia: 10, Romania: 4, Commonwealth of Independent States: 7, Italy: 3, Germany: 1, Hungary: 1, Northern Africa: 2, Iran: 1.

#### Ww-group

**Fig 2:**
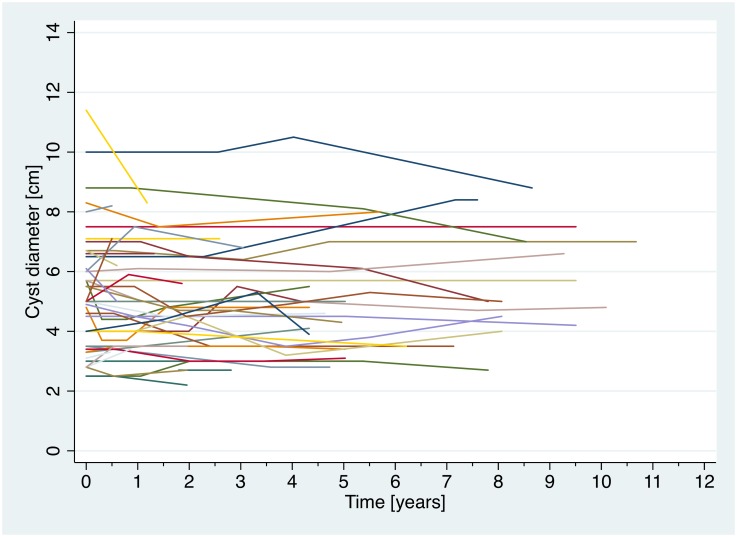
Stability of cyst diameters of ww-cysts. There is no substantial reduction in cyst diameter over time.

**Fig 3:**
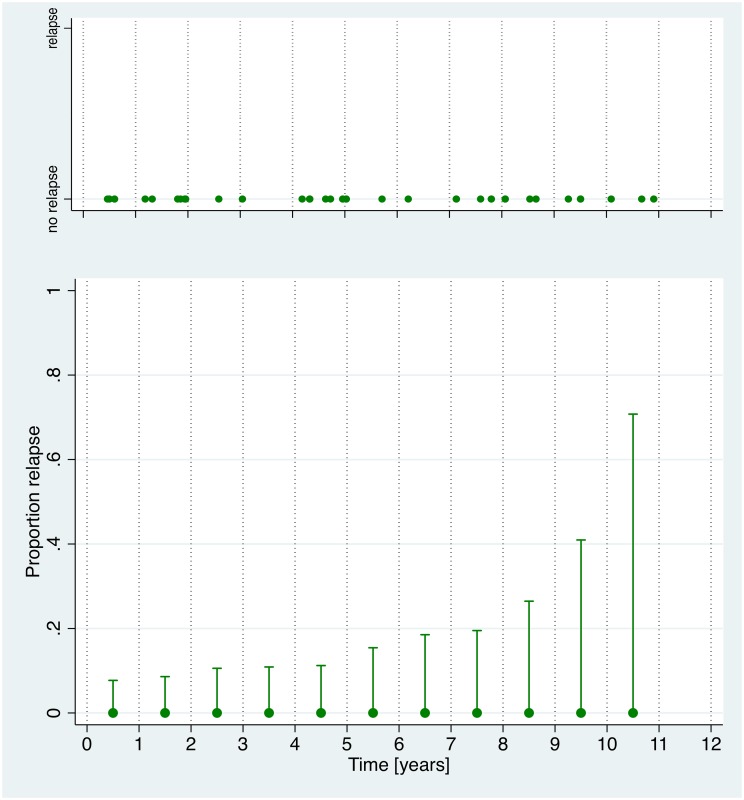
Ww-group relapses over time. No cysts showed relapse during the observation period. In the lower part of the figure confidence intervals of the proportion of relapse are shown. Uncertainty in the estimated proportion of relapse over time increases due to decreasing number of cysts under observation.

Forty-six cysts have been included in the analysis. All cysts were CE4 or CE5 at entry into the cohort. Forty-three cysts were localized in the liver; there was 1 splenic and 1 peritoneal cyst. Fig 2 shows stability of cyst diameter over time in ww-cysts. No relapses in ww-group cysts were observed (Fig 3).

#### Med-group

**Fig 4:**
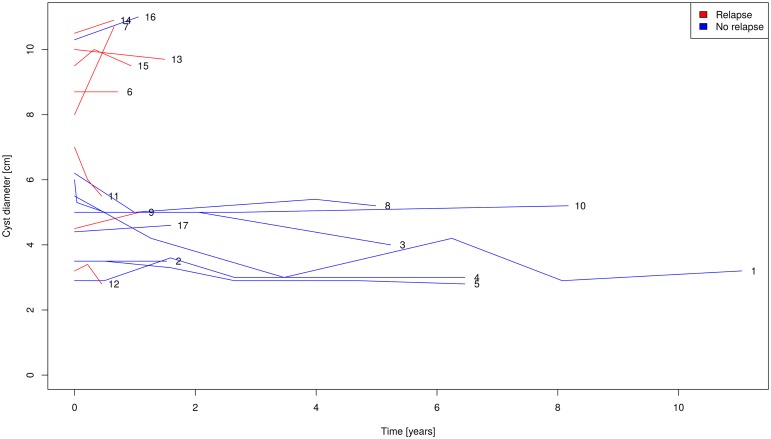
Cyst diameter over time in the med-group.

**Fig 5:**
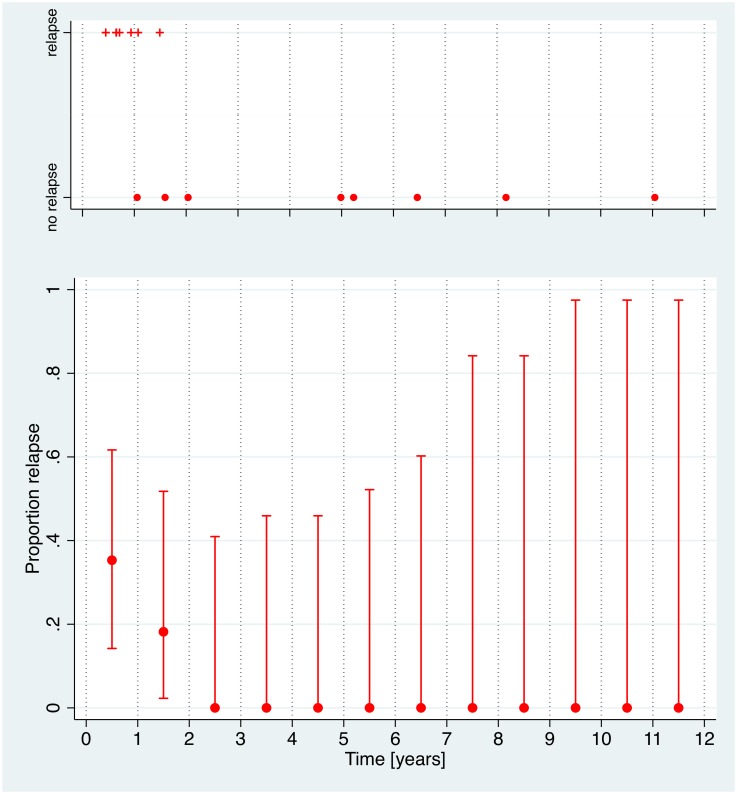
Med-group relapse over time.

Seventeen cysts were included in the med-group, 8 of the 17 analyzed cysts showed relapse. Regardless of cyst stage before treatment all cysts except one relapsed to stage CE3b. All cysts without relapse reached cyst stage CE4 after treatment with benzimidazoles. Cyst stage, localization and diameter at the beginning and end of medical treatment and at relapse are displayed in Tables 1 and 2. Fig 4 shows stability of cyst diameters of med-group cysts without relapse during the observation period. Confidence intervals for the proportion of relapse within 1-year time windows become wider each year due to decreasing number of cysts under observation (Fig 5). To minimize error for the determination of the time from inactivity to relapse only patients with ≤ 18 months between the visit when the relapse was detected and the previous visit were included into the analysis. 3 cysts with relapse and longer intervals from relapse to previous visit were not included into the analysis (Table 1). Among the cysts which were considered for the analysis, the mean time gap between these two observation time points (time of relapse and time of previous visit) was 0.71 years (95% CI 0.36–1.06).

**Table 1:** Cyst stage, diameter in cm and localization of med-patients with relapse. WHO cyst stage at the beginning of treatment, after treatment and at relapse.

Cyst ID	Cyst stage/diameter before R_x_*	Cyst stage/diameter after R_x_*	Cyst stage/diameter at relapse	Cyst localization
**6**	CE3b / 8,7	CE 4 / 8,7	CE3b / 8,7	liver
**7**	CE2 / 8	CE 4 / 8	CE3b / 10,7	liver
**9**	CE2 / 4,7	CE 4 / 4,5	CE3b / 5	liver
**11**	CE3a / 6	CE 4 / 7	CE3a / 5,5	liver
**12**	CE3b / 3,5	CE 4 / 3,2	CE3b / 2,8	liver
**13**	CE1 / 16	CE 4 / 10	CE3b / 9,7	liver
**14**	CE3b / 12	CE 4/ 10,5	CE3b / 10,9	spleen
**15**	CE3b / 10	CE 4 / 9,5	CE3b / 9,5	liver
^†^	CE3b / 3,3	CE 5 / 3,3	CE3b / 3,6	liver
^†^	CL / 7	CE 4 / 4	CE3b / 3,5	abdominal wall
^†^	CE3b / 6,5	CE 4 / 5	CE3b / 6,7	liver

* R_x_—treatment

^†^ excluded from study because observation gap from relapse to previous clinic visit was >18 months.

**Table 2:** Cyst stage, diameter in cm and localization of med-patients without relapse. WHO cyst stage at the beginning of treatment, after treatment and at the end of follow up.

Cyst ID	Cyst stage/diameter before R_x_*	Cyst stage/diameter after R_x_*	Cyststage/diameter end of follow up	Cyst localization
**1**	CE3b/ 5	CE4/ 5,5	CE4/ 3,2	liver
**2**	CE2/ 5	CE4/ 3,4	CE4/ 2,9	liver
**3**	CE1/ 3,9	CE4/ 5	CE4/ 4	liver
**4**	CE3a/ 3,8	CE4/ 2,9	CE4/ 3	liver
**5**	CE3a/ 3,8	CE4/ 3,5	CE4/ 2,8	lung
**8**	CE3b/ 6,5	CE4/ 6,2	CE4/ 5,2	liver
**10**	CE3b/ 5,1	CE4/ 6	CE4/ 5,2	lung
**16**	CE2/ 12	CE4/ 10,3	CE4/ 11	liver
**17**	CE2/ 4,5	CE4/ 4,4	CE4/ 4,6	liver

* R_x_-treatment.

**Fig 6:**
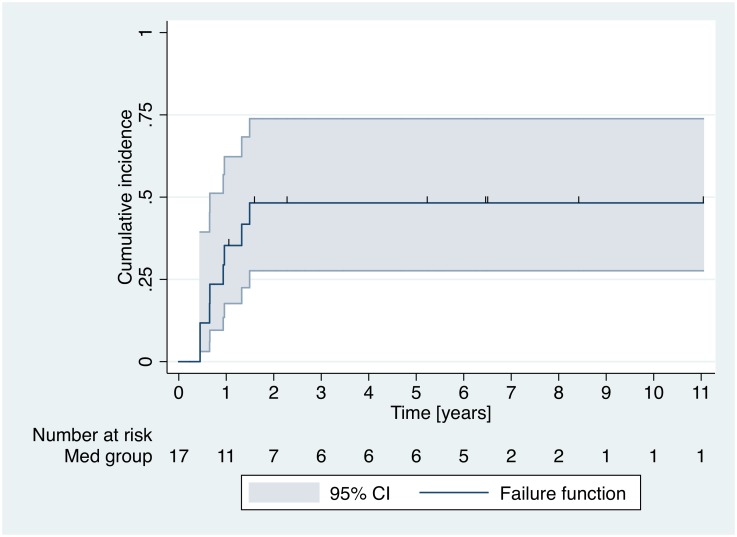
Kaplan-Meier Analysis of time to relapse.

Kaplan-Meier-Analysis of time to relapse in the med-group shows a cumulative incidence of relapse of around 50% within 18 months (Fig 6).

## Discussion

The benefits of cyst staging-based management of patients with cystic echinococcosis are not yet fully explored. Very obvious questions are: Do inactive cysts (CE 4 and CE 5) need treatment and is there a difference between cysts which reach CE4 and CE5 naturally or by benzimidazole therapy? So far only two CE clinical centers systematically explored the watch and wait approach, the Centre at the Pavia University Hospital [7,8] and our study point in the same direction. Since transition of active and transitional to inactive cysts is very slow and a matter of many years analysis of relevant outcomes such as relapse rates is biased by often big losses to follow-up. Due to a recall system overall loss to follow up in our patient cohort is small 15.5% (7/45) (Fig 1).

In the ww-group data impressively show how stable naturally inactivated cysts are. In the first 3–5 years of observation confidence intervals predicting relapse are narrow. The later increase is due to the decreasing number of cysts under observation and must be judged by biological plausibility. Over time inactive cysts increasingly consolidate and calcify making relapse less and less likely. Our data strongly support the watch and wait approach in patients with uncomplicated asymptomatic cysts in anatomical sites where they do not compromise vital structures such as vessels. Based on our data we suggest that a minimum follow-up of 3–5 years is sufficient in patients with naturally inactivated cysts.

**Fig 7:**
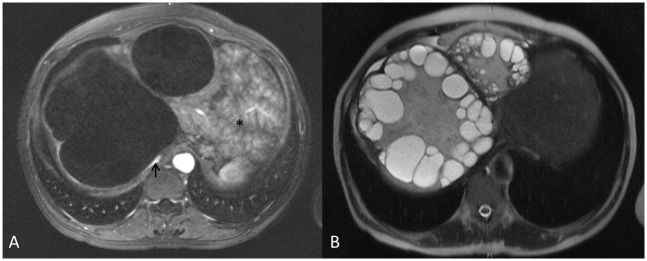
A: contrast enhanced MRI with compression of caval vein (arrow) and inhomogeneous perfusion of liver tissue (pseudo-Budd-Chiari syndrome, asterisk); B: T2-weighted MRI displaying solid and liquid components of CE 3b cysts.

In contrast cysts which reach inactivity through treatment with benzimidazoles behave differently. The relapse rate of 50% shown in the Kaplan-Meier analysis (Fig 6) corresponds well with the 2-year relapse rate of 60% in a previous study on benzimidazole treatment with cysts < 6cm in diameter responding better than larger cysts[9]. Regardless of cyst stage at the start of benzimidazole therapy those cysts which inactivate and relapse after discontinuation of drug treatment relapse to cyst stage CE3b with only 1 exception. In our cohort we observed relapses mainly in the first 18 months after treatment was stopped. Delayed detection of relapse in the med-group lead to the exclusion of three cysts from the analysis. We kept the error of determining this important time period as small as possible by excluding patients from analysis with ≥ 18 months between the visit when the relapse was detected and the previous visit when the cyst was still inactive. We would nevertheless suggest that cysts that were inactivated by medical treatment should be followed up for five years after stopping drug therapy. This also in view of the fact that most cysts relapse to CE3b, a particularly problematic cyst stage, which is difficult to treat medically [9]. It has also substantial growth potential to cause severe pathology; e.g. compromise of vital structures such as large vessels (Fig 7).

The small sample size is a limitation of the study; however, the results relapse after w&w only and relapse after medical treatment are very robust.

In conclusion, a substantial proportion of patients can be spared from any treatment through cyst staging. Cysts which inactivated through a natural course of involution do not relapse with very high likelihood. We recommend follow up of 5 years to confirm the stability of the inactive stage. Cysts driven into inactivity instead need careful monitoring to identify those which reactivate (around 50% within 18 months). 5 years follow-up after reaching an inactive stage appears safe to make a final decision on the need for further monitoring.

## Supporting Information

S1 Checklist STROBE Checklist. (DOCX)
