# Supplementary material for: Watch and Wait Management of Inactive Cystic Echinococcosis – Does the Path to Inactivity Matter – Analysis of a Prospective Patient Cohort
Source: PLoS Negl Trop Dis. 2016 Dec 19;10(12):e0005243. doi: 10.1371/journal.pntd.0005243 (PMC5207761; doi:10.1371/journal.pntd.0005243)
Supplement: S1 Checklist — (DOCX) [file pntd.0005243.s001.docx]

STROBE Statement—checklist of items that should be included in reports of observational studies

|  | Item No. | Recommendation | Page  No. | Relevant text from manuscript |
| --- | --- | --- | --- | --- |
| **Title and abstract** | 1 | (*a*) Indicate the study’s design with a commonly used term in the title or the abstract | 1 | Prospective patient cohort |
|  |  | (*b*) Provide in the abstract an informative and balanced summary of what was done and what was found | 2 | The event of interest “relapse” was defined as the transition of a cyst from an inactive stage (CE4, CE5) back to a transitional (CE3a, CE3b) or active stage (CE1, CE2). The watch & wait (ww) group included 30 patients with 46 inactive cysts who never received medical treatment. The benzimidazole-treated (med) group included 15 patients with 17 cysts. Data from the watch & wait group impressively show how stable naturally inactivated cysts are in contrast to cysts which reach inactivity through treatment with benzimidazoles. |
| Introduction | | | |  |
| Background/rationale | 2 | Explain the scientific background and rationale for the investigation being reported | 4 | Overdiagnosis and overtreatment are increasingly discussed in medical practice not surprisingly also for patients with cystic echinococcosis (CE). In neglected tropical diseases (NTD) this is particularly relevant since most patients are under the care of health services with limited resources where the risk of therapy induced complications are often disproportionate to the benefit of interventions. In high-tech environments conditions which would never have surfaced and bothered a person regularly get unintentionally diagnosed and unnecessarily treated |
| Objectives | 3 | State specific objectives, including any prespecified hypotheses | 4 | hypotheses: (a) uncomplicated active (CE1 and CE2) or transitional (CE3a and CE3b) CE cysts which progressed without treatment to stage CE 4 and CE 5 do not reactivate and can safely be left untreated, whereas (b) if these cyst stages are induced by albendazole (ABZ) treatment relapse is common and patients need to be followed-up long enough to not miss it. |
| Methods | | | |  |
| Study design | 4 | Present key elements of study design early in the paper | 5 | Patient data is collected prospectively, at every visit of CE patients a defined set of cyst and treatment data are entered into the CE database. |
| Setting | 5 | Describe the setting, locations, and relevant dates, including periods of recruitment, exposure, follow-up, and data collection | 4/5 | The Section of Clinical Tropical Medicine at Heidelberg University Hospital runs an interdisciplinary clinic for echinococcosis in cooperation with the Department of Diagnostic and Interventional Radiology with weekly radiological conferences, the Department of Surgery, the Department of Thoracic Surgery, the Interdisciplinary Endoscopy Center, and the Department of Parasitology since 1999. Our unit is a national clinical center for echinococcosis. |
| Participants | 6 | (*a*) *Cohort study*—Give the eligibility criteria, and the sources and methods of selection of participants. Describe methods of follow-up | 5/6 | 1. Watch & wait (ww) group.  Patients with single or multiple (maximum 8) inactive CE4 or CE5 cysts who never received medical treatment for CE.  2. Medical treatment (med) group  Patients with single or multiple (maximum 8) active CE cysts which were treated with benzimidazoles for CE and became inactive.  Patients were followed-up by ultrasound or magnetic resonance imaging.  A patient was considered lost to follow-up if he did not complete a minimum follow-up of 5 years or the last follow-up visit was > 24 months ago. Patients with disseminated CE were excluded. |
| Variables | 7 | Clearly define all outcomes, exposures, predictors, potential confounders, and effect modifiers. Give diagnostic criteria, if applicable | 6 | The event of interest “relapse” was defined as the transition of a cyst from an inactive stage (CE4, CE5) back to an active or transitional stage (CE1, CE2, CE3).  Proportion of cyst relapse in the med-group was calculated for each follow-up year since reaching the inactive stage.  We analyzed time to relapse using the Kaplan-Meier method. Cumulative incidence of relapse was calculated as one minus the Kaplan-Meier estimate |
| Data sources/ measurement | 8* | For each variable of interest, give sources of data and details of methods of assessment (measurement). Describe comparability of assessment methods if there is more than one group | *5* | The database was screened for patients with ww management or medical treatment and all follow-up data of patients eligible for the study were updated in June 2016. |
| Bias | 9 | Describe any efforts to address potential sources of bias | 6 | To determine the time to relapse with sufficient accuracy, relapse from CE4 and CE5 to a transitional (CE3a, CE3b) or active cyst stage (CE1, CE2) was only included into the analysis if the time between the visit when the cyst was still inactive and the visit when it relapsed was ≤ 18 months. |
| Study size | 10 | Explain how the study size was arrived at | 6 | Consecutive patients over a defined period of time |

Continued on next page

| Quantitative variables | 11 | Explain how quantitative variables were handled in the analyses. If applicable, describe which groupings were chosen and why |  | N/A |
| --- | --- | --- | --- | --- |
| Statistical methods | 12 | (*a*) Describe all statistical methods, including those used to control for confounding | 6/7 | Proportion of cyst relapse in the med-group was calculated for each follow-up year since reaching the inactive stage. The nominator consists of the number of cysts with relapse during the respective time window, the denominator is the number of cysts still under observation which are at risk for relapse. A binomial exact 95% confidence interval was calculated for each follow-up year.  In order to account for censoring we performed a time-to-event analysis for the med group. The start of the time at risk for each cyst was defined as the date of the visit when the inactive stage was diagnosed. In case medical treatment was still ongoing at the time of the visit, the time at risk started at the end of therapy. If this date did not coincide with a visit to the clinic, the cyst stage from the last visit was considered (last observation carried forward).  We analyzed time to relapse using the Kaplan-Meier method. Cumulative incidence of relapse was calculated as one minus the Kaplan-Meier estimate.  All analyses were performed using Stata Version 13.1 (StataCorp) and R software version 3.3.1 ([6](#_ENREF_6)). |
|  |  | (*b*) Describe any methods used to examine subgroups and interactions |  | N/A |
|  |  | (*c*) Explain how missing data were addressed | 7 | In case medical treatment was still ongoing at the time of the visit, the time at risk started at the end of therapy. If this date did not coincide with a visit to the clinic, the cyst stage from the last visit was considered (last observation carried forward). |
|  |  | (*d*) *Cohort study*—If applicable, explain how loss to follow-up was addressed | 6 | A patient was considered lost to follow-up if he did not complete a minimum follow-up of 5 years or the last follow-up visit was > 24 months ago. |
|  |  | (*e*) Describe any sensitivity analyses |  | N/A |
| Results | | | | |
| Participants | 13* | (a) Report numbers of individuals at each stage of study—eg numbers potentially eligible, examined for eligibility, confirmed eligible, included in the study, completing follow-up, and analysed | 7 | 45 patients of the Heidelberg CE patient cohort fulfilled the inclusion criteria. 30 patients with 46 cysts were included in the ww-group, and 15 patients with 17 cysts into the med-group. The mean age of ww-patients was 41.5 (8-69), 18 patients were male, 12 patients female, mean follow-up for ww-patients was 5.4 (0.5-10.9) years. 6 patients were lost to follow-up in the ww-group. The mean age of med-patients was 35.8 (13-65), 4 patients were male, 11 patients female, mean follow-up of med-patients was 7.3 (1.5-11.8) years. 1 patient was lost to follow-up in the med-group. |
|  |  | (b) Give reasons for non-participation at each stage |  | N/A |
|  |  | (c) Consider use of a flow diagram | 7 | Figure 1 |
| Descriptive data | 14* | (a) Give characteristics of study participants (eg demographic, clinical, social) and information on exposures and potential confounders | 7 | 45 patients of the Heidelberg CE patient cohort fulfilled the inclusion criteria. 30 patients with 46 cysts were included in the ww-group, and 15 patients with 17 cysts into the med-group. The mean age of ww-patients was 41.5 (8-69), 18 patients were male, 12 patients female, mean follow-up for ww-patients was 5.4 (0.5-10.9) years. 6 patients were lost to follow-up in the ww-group. The mean age of med-patients was 35.8 (13-65), 4 patients were male, 11 patients female, mean follow-up of med-patients was 7.3 (1.5-11.8) years. 1 patient was lost to follow-up in the med-group. Patients were from the following countries: 16 Turkey, 10 countries of the former Yugoslavia, 4 Romania, 7 Commonwealth of Independent States , 3 Italy, 1 Germany, 1 Hungary, 2 Northern Africa, 1 Iran.  WW-group  46 cysts have been included in the analysis. All cysts were CE4 or CE5 at entry into the cohort. 43 cysts were localized in the liver, there was 1 splenic and 1 peritoneal cyst.  17 cysts were included in the med-group, 8 of the 17 analyzed cysts showed relapse. Regardless of cyst stage before treatment all cysts except one relapsed to stage CE3b. All cysts without relapse reached cyst stage CE4 after treatment with benzimidazoles. |
|  |  | (b) Indicate number of participants with missing data for each variable of interest | 9/10 | Table 1and Table 2 |
|  |  | (c) *Cohort study*—Summarise follow-up time (eg, average and total amount) | 7 | mean follow-up for ww-patients was 5.4 (min-max: 0.5-10.9) years  mean follow-up of med-patients was 7.3 (min-max: 1.5-11.8) years |
| Outcome data | 15* | *Cohort study*—Report numbers of outcome events or summary measures over time | 8 | No cysts showed relapse during the observation period. In the lower part of the figure confidence intervals of the proportion of relapse are shown. Uncertainty for the prediction of relapse over time increases due to decreasing number of cysts under observation.  17 cysts were included in the med-group, 8 of the 17 analyzed cysts showed relapse. Among the cysts which were considered for the analysis, the mean time gap between these two observation time points (time of relapse and time of previous visit) was 0.71 years (95% CI 0.36 – 1.06).  Kaplan-Meier-Analysis of time to relapse in the med-group shows a cumulative incidence of relapse of 51.8% (95% CI 26.2 - 72.4) after 1.5 years (Fig 6). |
| Main results | 16 | (*a*) Give unadjusted estimates and, if applicable, confounder-adjusted estimates and their precision (eg, 95% confidence interval). Make clear which confounders were adjusted for and why they were included | 9  10 | WW-group:  […]  No cysts showed relapse during the observation period. In the lower part of the figure confidence intervals of the proportion of relapse are shown. Uncertainty in the estimated proportion of relapse over time increases due to decreasing number of cysts under observation.  Med group:  […]  Kaplan-Meier-Analysis of time to relapse in the med-group shows a cumulative incidence of relapse of 51.8% (95% CI 26.2 - 72.4) after 1.5 years (Fig 6). |
|  |  | (*b*) Report category boundaries when continuous variables were categorized |  | N/A |
|  |  | (*c*) If relevant, consider translating estimates of relative risk into absolute risk for a meaningful time period |  | N/A |

Continued on next page

| Other analyses | 17 | Report other analyses done—eg analyses of subgroups and interactions, and sensitivity analyses |  | N/A |
| --- | --- | --- | --- | --- |
| Discussion | | | | |
| Key results | 18 | Summarise key results with reference to study objectives | 13 | In the ww-group data impressively show how stable naturally inactivated cysts are. In contrast cysts which reach inactivity through treatment with benzimidazoles behave differently. The relapse rate of 50% shown in the Kaplan-Meier analysis (Fig. 6) corresponds well with the 2-year relapse rate of 60% in a previous study on benzimidazole treatment with cysts < 6cm in diameter responding better than larger cysts([8](#_ENREF_8)). |
| Limitations | 19 | Discuss limitations of the study, taking into account sources of potential bias or imprecision. Discuss both direction and magnitude of any potential bias | 14 | The small sample size is a limitation of the study, however, the results relapse after W&W only and relapse after medical treatment are very robust. |
| Interpretation | 20 | Give a cautious overall interpretation of results considering objectives, limitations, multiplicity of analyses, results from similar studies, and other relevant evidence | 13 | In the ww-group data impressively show how stable naturally inactivated cysts are. In the first 3-5 years of observation confidence intervals predicting relapse are narrow. The later increase is due to the decreasing number of cysts under observation and must be judged by biological plausibility. Over time inactive cysts increasingly consolidate and calcify making relapse less and less likely. Our data strongly support the watch and wait approach in patients with uncomplicated asymptomatic cysts in anatomical sites where they do not compromise vital structures such as vessels. |
| Generalisability | 21 | Discuss the generalisability (external validity) of the study results | 14 | a substantial proportion of patients can be spared from any treatment through cyst staging. Cysts which inactivated through a natural course of involution do not relapse with very high likelihood. We recommend follow up of 5 years to confirm the stability of the inactive stage. Cysts driven into inactivity instead need careful monitoring to identify those which reactivate (around 50% within 18 months). 5 years follow-up appears safe to make a final decision on the need for further monitoring. |
| Other information | |  | | |
| Funding | 22 | Give the source of funding and the role of the funders for the present study and, if applicable, for the original study on which the present article is based |  | none |
